# Calcium phosphate ceramic as a model for enamel substitute material in dental applications

**DOI:** 10.1038/s41405-023-00152-w

**Published:** 2023-09-03

**Authors:** Phakvalunch Rujiraprasert, Sarat Suriyasangpetch, Anucharte Srijunbarl, Thawanrat Singthong, Chalermkwan Makornpan, Katanchalee Nampuksa, Thanaphum Osathanon, Dusit Nantanapiboon, Naruporn Monmaturapoj

**Affiliations:** 1 Department of Operative Dentistry, Faculty of Dentistry, Chulalongkorn University, Bangkok, Thailand; 2 Department of Advanced General Dentistry, Faculty of Dentistry, Mahidol University, Bangkok, Thailand; 3 Dental Materials Research and Development Center, Faculty of Dentistry, Chulalongkorn University, Bangkok, Thailand; 4 Assistive Technology and Medical Devices Research Center (A-MED), National Science and Technology Development Agency, Pathum Thani, Thailand; 5 Dental Stem Cell Biology Research Unit and Department of Anatomy, Faculty of Dentistry, Chulalongkorn University, Bangkok, Thailand

**Keywords:** Dental biomaterials, Calcium-based cement

## Abstract

**Objective:**

This study aimed to develop enamel substitute material using a mechanochemical technique.

**Materials and Methods:**

Hydroxyapatite was synthesized with and without tricalcium phosphate under uniaxial pressing of 10 and 17 MPa (HA10, HA17, BCP10, and BCP17), followed by sintering at 1250 °C for 2 h. Human enamel and dentin blocks were used as control groups. The mechanical properties were determined by compressive strength test and Vickers microhardness. The data were analyzed with one-way ANOVA and LSD post-hoc test (*α* = 0.05). The phase formation and morphology of the specimens were characterized by X-ray diffraction (XRD) and scanning electron microscopy (SEM).

**Results:**

HA17 and HA10 had compressive strength values comparable to enamel and dentin, respectively (*p* > 0.05). The microhardness of all synthesized groups was significantly higher than that of tooth structures (*p* < 0.05). From the XRD graphs, only the hydroxyapatite peak was observed in the control and HA groups. SEM images showed homogeneous hydroxyapatite grains in all groups, while the BCP groups contained higher porosities.

**Conclusions:**

Both HA10 and HA17 are suitable for use as the inorganic part of dentin and enamel substitutes.

## Introduction

A human tooth comprises multiple layers of different substrates and compositions. First, an outer enamel layer contains up to 96% inorganic materials, which are mostly well-organised hydroxyapatite crystals. As a result, it is the hardest substance in the human body and highly resists to acidic and chemical food intake [1]. From these superior characteristics, the enamel is an excellent protective layer of the tooth. The next layer is dentin, which comprises inorganic materials, with hydroxyapatite crystals as the majority. The remaining dentin contents are organic substances, for example, collagen and water. Therefore, it has low mechanical strengths than enamel but high elasticity, which benefits masticatory force distribution.

In various fields of dental research, especially in vitro studies, an extracted human tooth is commonly used in specimen preparations to mimic clinical conditions. However, it is almost impossible to control the configurations, compositions, and structures of the tooth. The collected tooth comes from different patients with various diagnoses, such as dental caries, severe periodontitis, impaction and etc., which contribute to the destruction of the structures and cannot fully represent the sound tooth. Moreover, the patient’s age also affects the mineral composition. The extracted tooth from younger patients tends to have a thicker enamel layer than the elderly because of shorter function times. In addition, the younger dentin also has lower mineral precipitations than the elders, as in tertiary dentin or sclerotic dentin. Another consideration is the disinfection procedures before use. If the tooth is not properly cleaned, it could be a source of infection. Therefore, numerous attempts have been made to seek and develop tooth substitute materials for laboratory experiments and clinical studies.

In the past few decades, numerous researchers have been seeking appropriate materials for tooth substitutes with the goal of clinical practice. Bioceramic is the commonly used materials in orthopedics and dentistry, especially calcium phosphate ceramics (Ca-P) because of their chemical similarity to bone and tooth. The Ca-P ceramics are biocompatible and bioactive, having high cell adhesion, cell proliferation, and new bone formation [2–4]. Hydroxyapatite belongs to one of these Ca-P groups, which is mainly composed of calcium and phosphorus. It is widely used as a bone substitute because of its excellent biocompatibility over other bioceramics. Despite the high degree of hardness, it has limited resorbability and bone remodeling process, which is quite different from a human tooth. Therefore, tricalcium phosphate (TCP), which has a higher biodegradability, is used to mix with hydroxyapatite to replace the bone and mimic the tooth’s properties. In order to control the resorbability and biological properties of the materials, a mixture of hydroxyapatite and TCP, so-called biphasic calcium phosphate (BCP) was introduced and studied to be used as bone and teeth substitute materials.

Currently, there are five sintering methods for hydroxyapatite synthesis, that is, high-temperature solid-state synthesis, aqueous phase synthesis, hydrothermal process, synthesis using molten salts, and using gels [5]. The most common method is sintering with high temperatures because it produces highly packed and good mechanical properties of synthesis hydroxyapatite. Consequently, most studies focused on the effect of sintering temperature on the mechanical properties of hydroxyapatite rather than concentrated on the mixture of hydroxyapatite and TCP, so-called BCP. However, there are a few studies regarding the effect of pressure stress in the formation of hydroxyapatite and BCP specimens. Furthermore, there has still been no ideal material that achieves all the chemical compositions and mechanical properties of the human tooth.

Thus, in this study, we proposed the optimal process for tooth substitute materials. Both hydroxyapatite and BCP were synthesized using a mechanochemical technique. Then the as-synthesis powders were fabricated using a uniaxial pressing method under the pressure of 10 or 17 MPa, followed by sintering at 1250 °C for 2 h. The mechanical properties of the packed samples were determined in comparison to those of human enamel and dentin as controls.

## Materials and methods

### Hydroxyapatite, tricalcium phosphate (TCP), and biphasic calcium phosphate (BCP) preparation

#### Hydroxyapatite and TCP powder synthesis

The starting materials in this study were CaCO_3_ (Fluka Chemie GmbH, Buchs, Switzerland) and CaHPO_4_ (Sigma Aldrich Chemie GmbH, München, Germany) due to chemical stability and well distribution of particle sizes [4]. All reagents were analytical reagent grade and were used as received. Deionized water (DI) was used in all processing steps. The starting materials CaCO_3_ and CaHPO_4_ with Ca/P molar ratios of 1.67 and 1.5 were used to produce pure hydroxyapatite and TCP powder following Eqs. (1) and (2) via a mechanochemical method [6].

1 $$\left( {{{{{{{{\mathrm{for}}}}}}}}\,{{{{{{{\mathrm{hydroxyapatite}}}}}}}}} \right)\,2{{{{{{{\mathrm{CaCO}}}}}}}}_3 + 3{{{{{{{\mathrm{CaHPO}}}}}}}}_4\; = > \;{{{{{{{\mathrm{Ca}}}}}}}}_{10}\left( {{{{{{{{\mathrm{PO}}}}}}}}_4} \right)_6\left( {{{{{{{{\mathrm{OH}}}}}}}}} \right)_2 + {{{{{{{\mathrm{H}}}}}}}}_2{{{{{{{\mathrm{O}}}}}}}} + 2{{{{{{{\mathrm{CO}}}}}}}}_2$$

2 $$\left( {{{{{{{{\mathrm{for}}}}}}}}\,{{{{{{{\mathrm{TCP}}}}}}}}} \right)\quad \quad {{{{{{{\mathrm{CaCO}}}}}}}}_3 + {{{{{{{\mathrm{CaHPO}}}}}}}}_4 = > {{{{{{{\mathrm{Ca}}}}}}}}_3\left( {{{{{{{{\mathrm{PO}}}}}}}}_4} \right)_2 + {{{{{{{\mathrm{H}}}}}}}}_2{{{{{{{\mathrm{O}}}}}}}} + 2{{{{{{{\mathrm{CO}}}}}}}}_2$$

CaCO_3_ and CaHPO_4_ were mixed and then ball milled using zirconia balls in DI and ethanol as a medium for 48 h for the synthesis of hydroxyapatite and TCP, respectively. The particles were dried in a hot air oven at 100 °C for 24 h. The as-synthesized hydroxyapatite and TCP powder particle size was approximately 4.5 µm measured by Zetasizer (Malvern Panalytical, Malvern, UK).

#### Dense sample preparation

The as-synthesized hydroxyapatite powder and the mixture of as-synthesized hydroxyapatite and as-synthesized TCP at a ratio of 70:30, so-called biphasic calcium phosphate (BCP) powder, were compacted under a uniaxial pressing of 10 MPa (HA10, BCP10) and 17 MPa (HA17, BCP17) for a dwell time of 60 s to consolidate the specimens into a bar with dimensions of 16 × 5 × 2 mm for the compressive strength test. For the Vickers microhardness test, the pellets (13 mm diameter and 3 mm thickness) were fabricated. The green compact was sintered by a two-step heat treatment in a normal atmosphere at 400 °C with a 100 °C per hour heating rate and 2 h holding time. Then, the temperature was increased to 1250 °C with the same heating rate of 100 °C per hour and 2 h holding time, followed by a furnace cooling to room temperature.

### Human tooth preparation

The protocol was conducted in accordance with the Declaration of Helsinki and approved by the Human Research Ethical Committee of the Faculty of Dentistry, Chulalongkorn University (approval No. HREC-DCU 2022-075). Human premolars extracted according to patients’ treatment plans were collected. All teeth were inspected with a stereomicroscope (Stereo Microscope SZ61, Olympus, Tokyo, Japan) for cracks, decay, and restorations. The teeth were cleaned with a dental scaler and polished with fine pumice slurry using a low-speed handpiece before being stored in a 0.1% thymol solution at 37 °C for disinfection.

### Compressive strength test

**Fig. 1:**
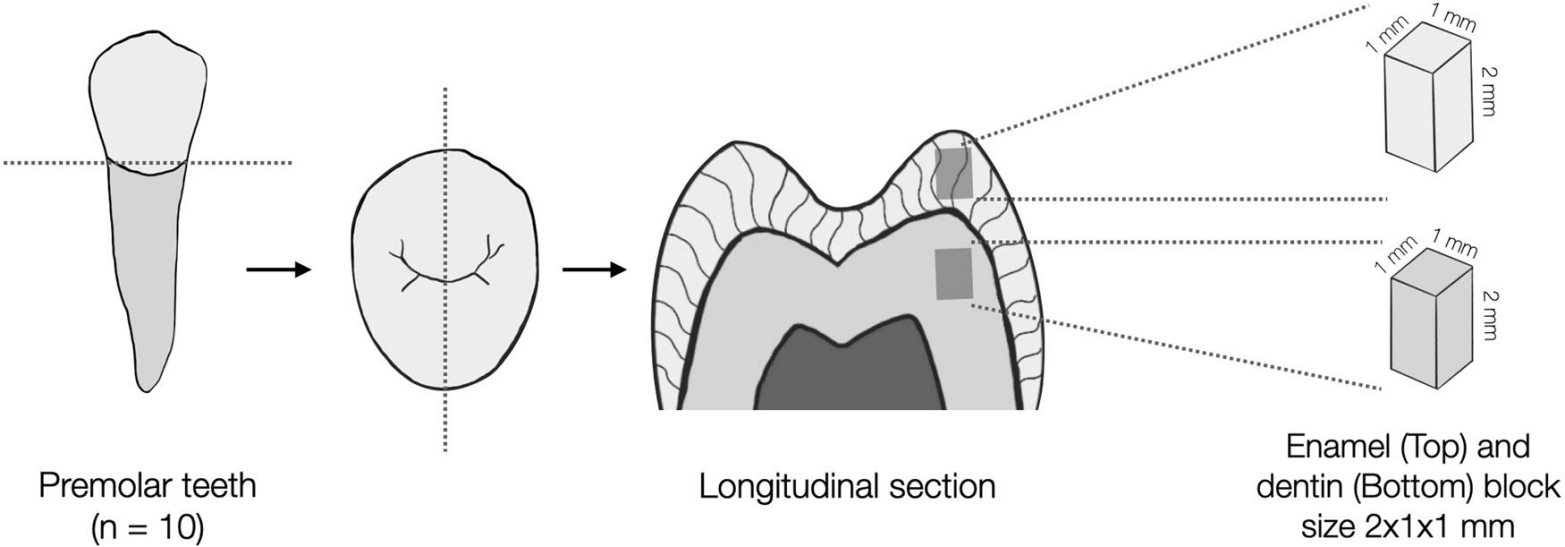
Tooth preparation diagram. Preparation of enamel and dentin block for the compressive strength test.

Only cusp tip areas in a coronal part of the tooth were used for the compressive strength test. The tooth was cut mesiodistally using a diamond saw blade (Isomet1000, Buehler, IL, USA) into ten enamel and dentin blocks with dimensions of 2 × 1 × 1 mm, which were confirmed by a digital caliper (CD-15AX, Mitutoyo, Kanagawa, Japan) before storage in 37 °C artificial saliva until use (Fig. 1).

**Fig. 2:**
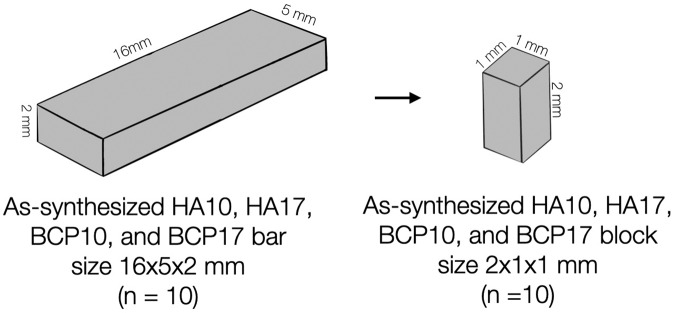
Hydroxyapatite block preparation. Preparation of hydroxyapatite blocks for the compressive strength test.

The hydroxyapatite bars were cut into 10 small blocks of 2 × 1 × 1 mm. dimensions, which were confirmed by a digital caliper (CD-15AX, Mitutoyo, Kanagawa, Japan) (Fig. 2). A universal testing machine (LR10K, LLOYD Instruments, Bognor Regis, UK) with a 1000 N load cell and 0.5 mm/min crosshead speed was used. The compressive strength was calculated following Eq. (3), where F is the compressive strength value (MPa), *P* is the maximum load until material failure (*N*), and A is the cross-sectional area of the material resisting the load (mm^2^).

3 $${{{{{{{\mathrm{F = }}}}}}}}\frac{{{{{{{{\mathrm{P}}}}}}}}}{{{{{{{{\mathrm{A}}}}}}}}}$$

### Vickers microhardness test

For the microhardness test, 10 teeth were cut in a longitudinal section and mounted in an acrylic resin block. Wet silicon carbide abrasive papers with 600, 1000, 1200-grit, and alumina oxide polishing paste with 0.05 μm particle size were used for polishing to a smooth flat surface. The specimens were stored in 37 °C artificial saliva until use.

**Fig. 3:**
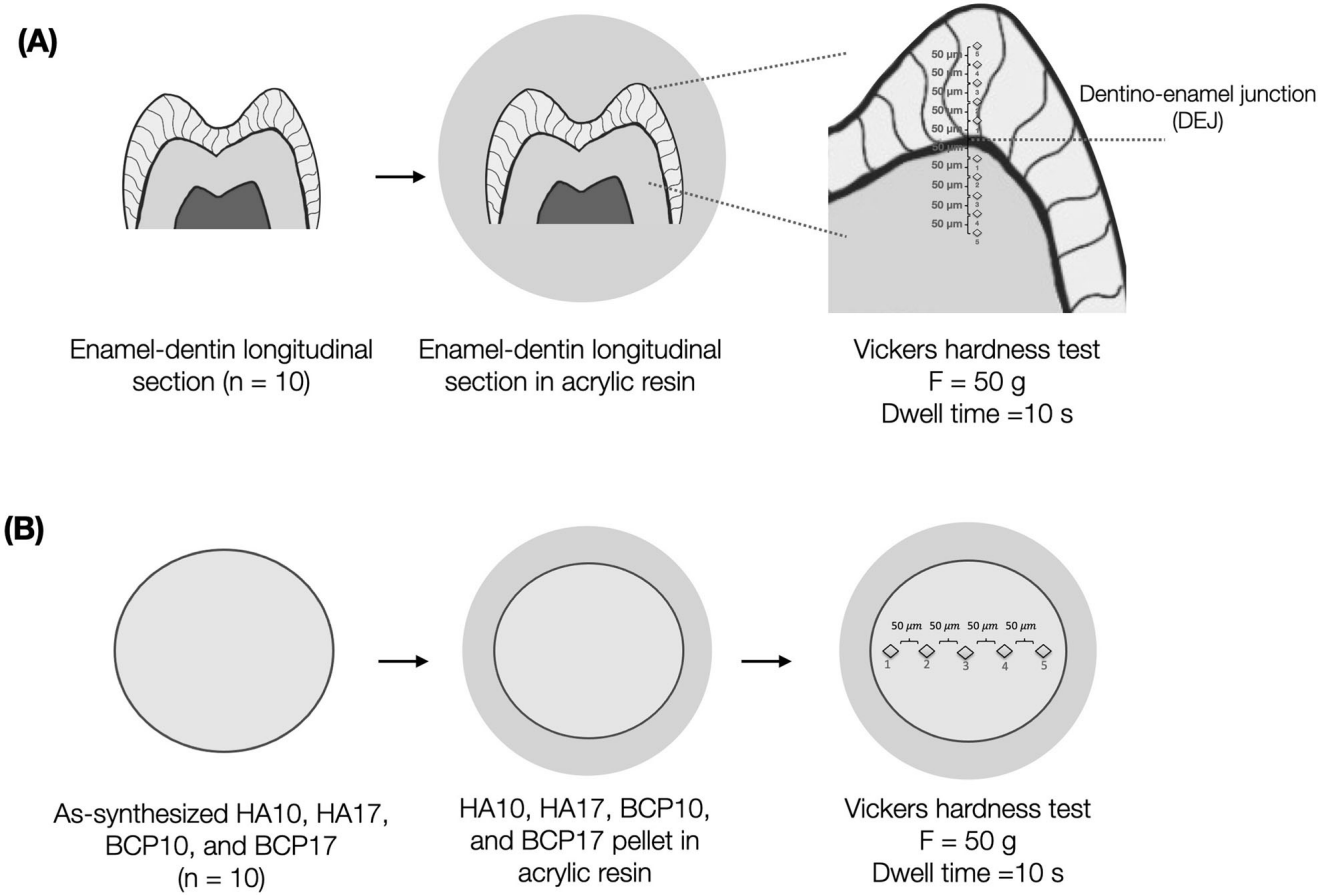
Vickers microhardness test diagram. Vickers microhardness testing of (**A**) enamel–dentin and (**B**) hydroxyapatite specimens.

A Vickers microhardness tester (FM-810, FUTURE-TECH, Kanagawa, Japan) with a 50-g load and 10 s dwell time was applied on the surfaces of the specimens. Five indentations with 50 μm apart were located both occlusally and apically from a dentino-enamel junction of the tooth (Fig. 3A). For the hydroxyapatite groups, five indentations were applied (Fig. 3B). The length of each two diagonals of the square-shaped indentation was immediately measured by an optical microscopy with a 300 magnification and ±1 µm measurement error.

### Specimen characterization using X-ray Diffraction (XRD) and scanning electron microscope (SEM)

The as-synthesized hydroxyapatite and as-synthesized TCP powders were analyzed phase formation by using X-ray Diffraction (XRD: PANalytical X’Pert Pro, PANalytical, Almelo, the Netherlands) with CuK_α_ radiation (K_α_ = 1.5406 Å) operating at 30 mA and 40 kV. XRD was performed from 20 to 60° 2θ, at a step size of 0.02° 2θ and a scanning speed of 2.4° 2θ/min with a CuKα target. The spectra were analyzed using JADE software and JCPDS cards. The amorphous phase of as-synthesized hydroxyapatite and as-synthesized TCP powders confirmed the occurrence of hydroxyapatite (JCPDS No. 09-0432), and β-tricalcium phosphate (β-TCP) (JCPDS No. 09-0619) by XRD pattern.

In the same manner, both sintered hydroxyapatite and BCP in the dense form samples were also undergone an XRD (SmartLab^®^, Rigaku, Tokyo, Japan) with CuK_α_ radiation (K_α_ = 1.5406 Å) operating at 30 mA and 40 kV from 20 to 60° 2θ, at a step size of 0.01° 2θ and at a scanning speed of 5° 2θ/min to confirm the formation of hydroxyapatite (JCPDS No. 09-0432) in HA10 and HA17 samples and the mixture of both hydroxyapatite (JCPDS No. 09-0432) and β-TCP (JCPDS No. 09-0619) in BCP10 and BCP17 samples. The proportions between hydroxyapatite and TCP in BCP samples were calculated by equations:

4 $${{{{{{{\mathrm{Hydroxyapatite}}}}}}}}\,\left( \% \right) = \frac{{{{{{{{{\mathrm{I}}}}}}}}_{{{{{{{{\mathrm{HA}}}}}}}}}}}{{{{{{{{{\mathrm{I}}}}}}}}_{{{{{{{{\mathrm{HA}}}}}}}}} + {{{{{{{\mathrm{I}}}}}}}}_{{{{{{{{\mathrm{TCP}}}}}}}}}}} \times 100$$

5 $${{{{{{{\mathrm{TCP}}}}}}}}\,\left( \% \right) = \frac{{{{{{{{{\mathrm{I}}}}}}}}_{{{{{{{{\mathrm{TCP}}}}}}}}}}}{{{{{{{{{\mathrm{I}}}}}}}}_{{{{{{{{\mathrm{HA}}}}}}}}} + {{{{{{{\mathrm{I}}}}}}}}_{{{{{{{{\mathrm{TCP}}}}}}}}}}} \times 100$$

The specimens were gold coated using a gold evaporation coating unit (JFC 1200, JEOL, Tokyo, Japan) before examining the morphology with a scanning electron microscope (Quanta250, FEI, USA). The SEM was operated at 20 kV and 10,000 magnification with a working distance optimized for imaging and large spot size.

### Statistical analysis

SPSS version 28.0 software (IBM, Chicago, IL, USA) was used for all statistical tests. *P*-values less than 0.05 were considered statistically significant differences. A Shapiro–Wilk test was used to determine the normality of mean differences in compressive strength and microhardness values. Data were normally distributed (*p* > 0.05). Mean differences were analysed by one-way ANOVA and LSD post-hoc test.

## Results

**Fig. 4:**
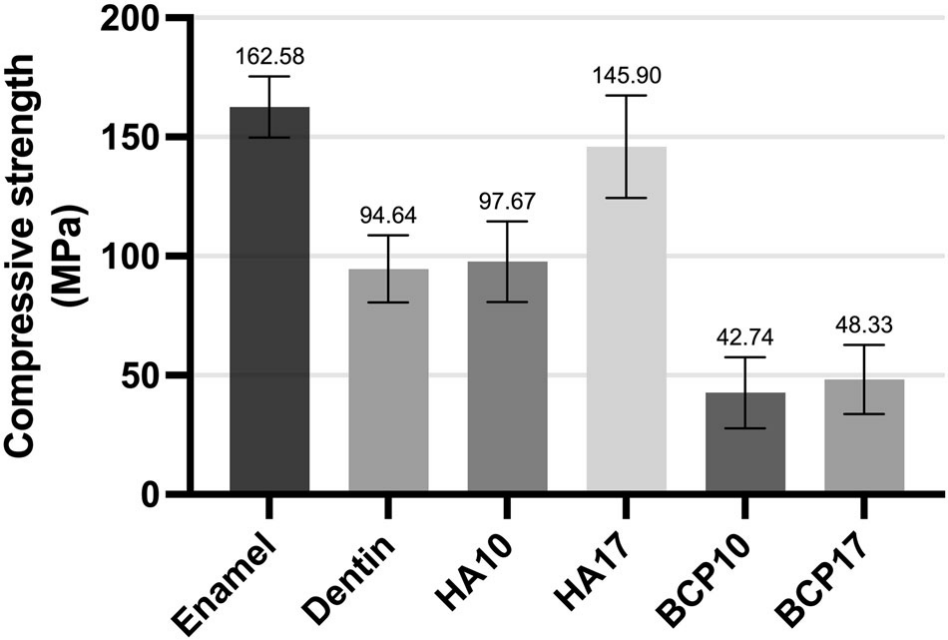
Compressive strength results. The bar chart represented the compressive strength of enamel, dentin, hydroxyapatite, and BCP samples.

The result of compressive strength was shown in Fig. 4. The highest compressive strength was from enamel, which did not show significant differences with HA17 (*p* > 0.05). The HA10 value was also not different with the dentin (*p* > 0.05), while both BCP groups were at half of the dentin. The increase in the uniaxial stress in the HA groups obviously resulted in almost twice the compressive strength value (*p* < 0.001). However, this was not observed in the BCP groups (*p* > 0.05).

**Fig. 5:**
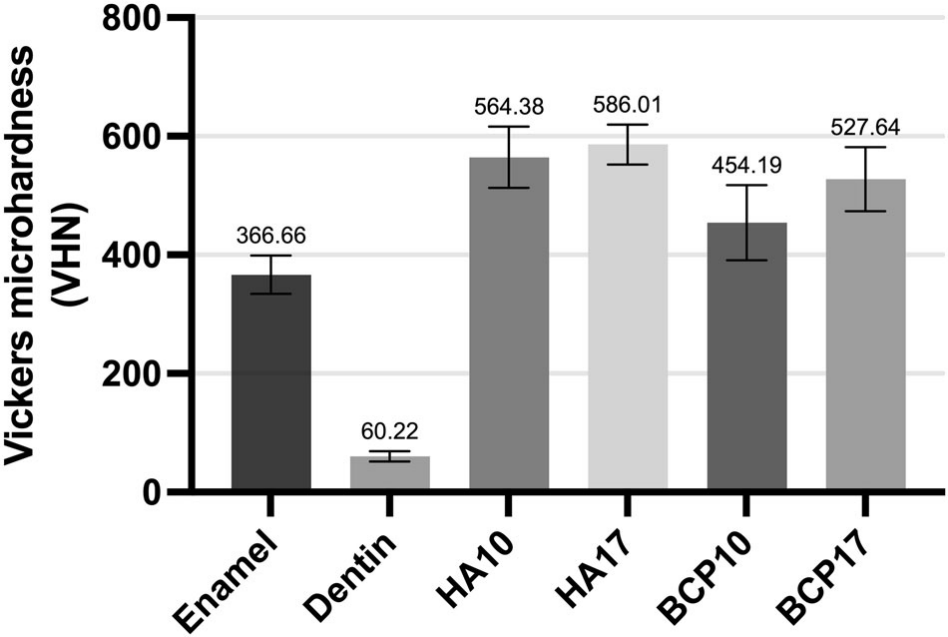
Vickers microhardness results. The bar chart represented the Vickers microhardness of enamel, dentin, hydroxyapatite, and BCP samples.

The result of microhardness was shown in Fig. 5. The Vickers microhardness of all HA and BCP groups was significantly greater than enamel and dentin (*p* < 0.001). The higher uniaxial stress barely affected the microhardness value of both HA and BCP groups (*p* > 0.05). Higher pressing pressure resulted in higher hardness values in all HA and BCP groups.

**Fig. 6:**
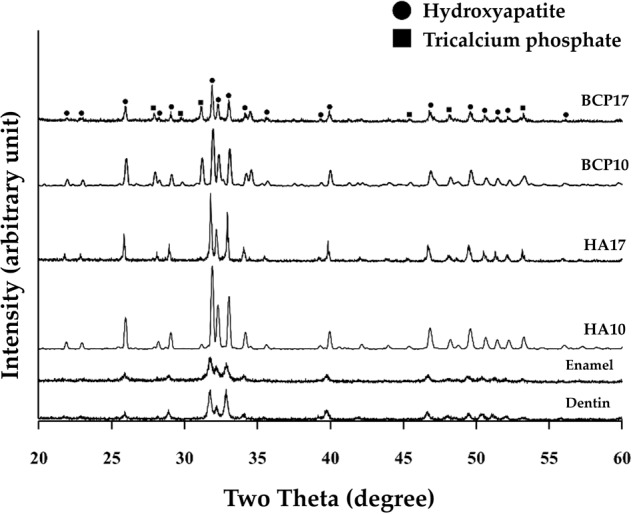
XRD results. The graph represented the XRD pattern of enamel, dentin, hydroxyapatite, and BCP samples.

For analyzing sample characteristics, the XRD revealed that the enamel and dentin were hydroxyapatite (JCPDS No. 09-0432) with no minor phases. There were also no minor phases in HA10 and HA17, and the XRD peaks matched the hydroxyapatite. In the BCP samples, hydroxyapatite (JCPDS No. 09-0432) and β-TCP (JCPDS No. 09-0619) were found as main phases with a HA/TCP ratio of 65:35 (Fig. 6).

**Fig. 7:**
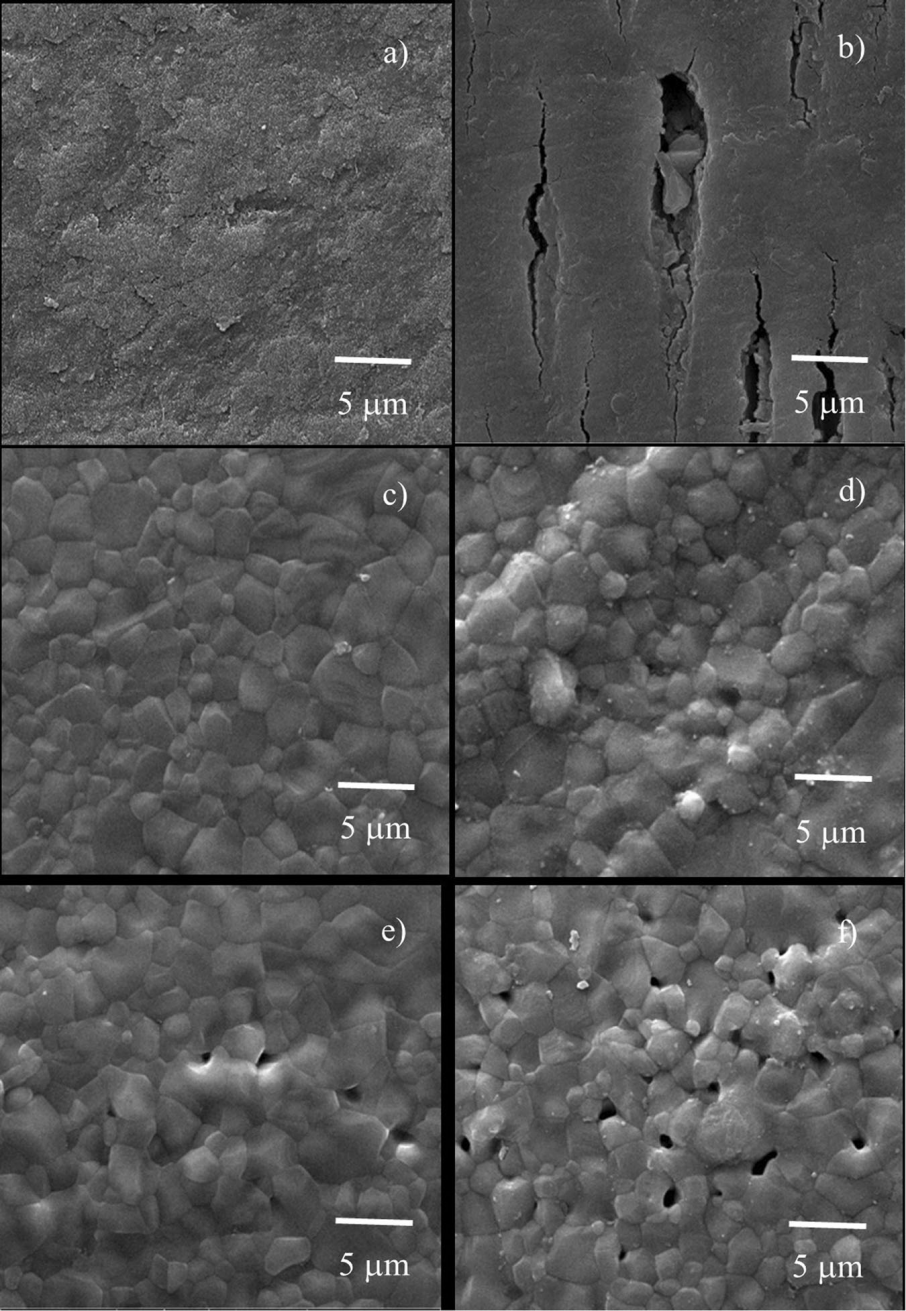
SEM images. The SEM micrographs at 10,000× of (**a**) enamel, (**b**) dentin, (**c**) HA10, (**d**) HA17, (**e**) BCP10, and (**f**) BCP17 samples.

The SEM images showed homogeneous hydroxyapatite on the enamel and dentin surfaces (Fig. 7). Both of the HA and BCP groups consisted of well-equiaxed hexagonal grains of 5 μm hydroxyapatite. However, BCP with higher uniaxial stress contained more porosities, which was not observed in the HA groups.

## Discussion

Recently, the biomimetic concept has gained attention in various fields of dentistry. For example, in implant dentistry, calcium phosphate, and hydroxyapatite are used in implant coatings for therapeutic effects and to improve osseointegration with alveolar bone [7]. In minimally invasive dentistry, remineralization is preferred in non-cavitated lesions rather than restorations to preserve tooth structure. In restorative dentistry, there have been numerous efforts to develop and synthesize the ideal restorative materials to replace the missing tooth structure over time. Synthetic materials should have chemical and mechanical properties similar to those of the human tooth, which can also benefit both in vitro and in vivo. Therefore, it can broaden the research fields while minimizing the ethical issues in using a natural tooth.

The hydroxyapatite in this study was synthesized from the mechanochemical technique. It is a commonly used method because of its high reproducibility and low manufacturing cost [8]. Sintering temperature is an essential factor that influences the strength of hydroxyapatite and BCP. Sintering at temperatures between 1000 and 1500 °C eliminates hydroxide functional groups (-OH) in the hydroxyapatite matrix because of dehydration. As a result, hydroxyapatite decomposes into β-TCP and tetra-calcium phosphate (TTCP) [9]. It is also reported that hydroxyapatite was transformed to β-TCP after sintering from 650 to 1200 °C [10]. Another previous study demonstrated that 1250 °C was the optimal sintering temperature that improved mechanical strengths, while 1300 °C decreased the strength [11]. However, sintering at a temperature of 1200 °C higher has led to the formation of β-TCP and TTCP [12].

Compositions and properties under two uniaxial pressures of both HA and BCP groups were evaluated, i.e., 10 and 17 MPa, at 1250 °C sintering temperature. The HA specimens were developed successfully with or without TCP. The TCP was added to the hydroxyapatite to lower the mechanical strength of the material, forming the BCP groups. HA17 and HA10 had similar compressive strength with enamel and dentin, respectively. The addition of TCP greatly reduced the microhardness and compressive strength compared to those of the non-TCP groups.

The compressive strength test is frequently used to determine the strength of the material as it represents the strength of the entire bulk of the material. The compressive strength values of enamel and dentin found in this study were 163 and 95 MPa. Only both HA groups had compressive strength similar to that of human tooth structures. However, the two groups of BCP showed considerably lower compressive strength values due to the addition of TCP minerals that produced porosities [13, 14]. It is worth mentioning that the decomposition of hydroxyapatite into β-TCP reduced the density, leading to the whole macrocracks [15]. The assumption was confirmed by the SEM images that exhibited micropores in the specimens.

The material hardness is attributed to a function of both grain sizes and porosities of the material [16]. However, the effect of grain sizes on hardness value was not observed in every ceramic material [17]. There was a study reported that the hardness decreased monotonically as the volume fraction porosity increased for essential ceramics [18]. In addition, hardness can be a function of the second phases that are present in the microstructure. The hardness values of enamel and dentin found in the study were 360 and 60 VHN, respectively, which were in agreement with other studies [19, 20]. The highest hardness values of the HA10 and HA17 compared to other samples, including the controls, could be explained by the absence of the β-TCP phase that lowered mechanical properties [11]. As a result, the β-TCP phase observed in the BCP groups attributes to the lower microhardness than the HA groups. Despite the comparable compressive strength between both the HA groups and the control groups, the microhardness values were still significantly high. If it were to be used as a restorative material, this would be an advantage in the oral cavity, especially in an acidic environment and in high caries-risk patients.

The XRD was used for characterization. Both the HA10 and HA17 groups exhibited only hydroxyapatite with no minor phases, which are similar to the enamel and dentin groups, despite differences in peak intensity. Higher hydroxyapatite implies higher crystallinity of the samples after sintering [21, 22]. However, in the BCP groups, this diffraction peak was observed along with the β-TCP being the major phases with hydroxyapatite/TCP ratio at approximately 65:35 without any minor phases. The ratio between hydroxyapatite and TCP was calculated by the proportion of the strongest intensity between hydroxyapatite and TCP that appeared at 31.8° and 31°, respectively (Eqs. 3 and 4)[6]. The partial transformation of hydroxyapatite to TCP is caused by the slight change in the hydroxyapatite/TCP ratio. The decomposition products can significantly influence the hardness and other mechanical properties of hydroxyapatite and BCP [23, 24]. However, this confirmed the suitable sintering temperature at 1250 °C due to no minor phases occurring after sintering.

The SEM images illustrated the homogeneous matrix of hydroxyapatite on the surfaces of enamel and dentin. Normally, the enamel is built from densely packed of fine rod-like carbonate apatite crystals with a rectangular cross-sectional area [25, 26]. Some small pores rather appeared in dentin than in enamel. It is very difficult to identify the grain size of both enamel and dentin. A slight etch with a mild acid on the surface of enamel and dentin could help to better understand their structures [25].

The HA and BCP groups under both pressuring stresses exhibited well-equiaxed hexagonal grains of hydroxyapatite with a 5 µm average grain size. It can be assumed that the increase in the pressuring stress did not affect the grain size of the HA and BCP groups. However, porosities were affected, as more porosities were observed with 17 MPa pressure. This could be due to higher densification [17]. Furthermore, TCP also created microporosities in BCP groups, compared to the others, which corresponded to the XRD results. The reason is that the decomposition of hydroxyapatite to TCP during the sintering process promotes the release of water from the molecules and results in surface porosities [27].

Despite the better properties mentioned above, the structure of our hydroxyapatite was different from that of human enamel. The fabrication process cannot fully replicate the entire enamel due to its complicated arrangements. The basic component of enamel is an enamel rod. These rods arrange linearly parallel to one another and perpendicular to the dentino-enamel junction (DEJ) and outer tooth surface [2]. At a higher structural level, these rod bundles decussate and appear as the Hunter-Schreger bands, which, to date, cannot be done in the laboratory. As a result, this unique hierarchical structure contributes to its outstanding mechanical properties [28]. In contrast, dentin has low inorganic minerals but high organic material, which mostly consists of type I collagenous collagen fibers [29]. Moreover, there are small canals called dentinal tubules, which are surrounded by peritubular dentin. The peritubular dentin can thicken with time because of continuing mineral deposition and its living connective tissue. Therefore, dentin is more challenging in the synthesis.

The limitation of this study is that the experiment did not mimic clinical situations, as it did not undergo artificial aging, such as thermocycling and water storage. Additionally, the compressive strength performed in the study was under a static condition, which differed from the dynamic chewing situation. Further studies should investigate other properties, such as elastic modulus and wear resistance, before using synthetic hydroxyapatite as tooth substitute materials.

## Conclusions

The hydroxyapatite and BCP (HA70:TCP30) ceramics were successfully synthesized through a uniaxial pressing technique under 10 and 17 MPa, followed by the sintering process. The HA10 and HA17 had nearly the same compressive strength as human dentin and enamel, respectively, despite the high microhardness values. Therefore, both HA10 and HA17 are suitable models for inorganic part of dentin and enamel substitutes.

## Data Availability

The data that support the findings of this study are available from the corresponding author, DN, upon reasonable request.
